# Supplementary material for: A novel SNF2 ATPase complex in Trypanosoma brucei with a role in H2A.Z-mediated chromatin remodelling
Source: PLoS Pathog. 2022 Jun 8;18(6):e1010514. doi: 10.1371/journal.ppat.1010514 (PMC9236257; doi:10.1371/journal.ppat.1010514)
Supplement: S1 Table — A Table. Identification of a new complex associated with HAT2. 10 proteins were identified via mass spectrometry in two Co-IP experiments. The initial Co-IP was performed with Tb927.11.11530, the reciprocal Co-IP with the protein Tb927.11.10070 was performed to confirm the Tb927.4.2000 co-IP data. Only Tb927.3.4140 could be identified in the initial but not in the reciprocal Co-IP experiment. The “Annotation” column indicates the curated annotation that was found for the corresponding accession number in the TriTyp database. The” identified domains” column displays the domains that were found by BLAST search using the NCBI database. The Phyre2 modelling column indicates proteins that were identified by homology modelling. Coverage (Cov.) indicates the coverage in percent between query and template. The confidence (Conf.) represents the relative probability in percent (from 0 to 100) that the match between query and template is a true homology. The nuclear enrichment score (NES) indicates a nuclear localization if positive. The last column shows in which of the two Co-IPs the protein could be identified. B Table. Identification of a new complex associated with HAT1. 11 proteins were identified via mass spectrometry in two co-IP experiments. The initial Co-IP was performed with Tb927.9.2910, the reciprocal Co-IP with the protein Tb927.1.650 was performed to confirm the Tb927.9.2910 co-IP data. Tb927.6.1240, Tb927.10.8310 and Tb927.10.9930 could only be identified in the initial but not in the reciprocal co-IP experiments. The “Annotation” column indicates the curated annotation that was found for the corresponding accession number in the TriTyp database. Proteins labelled in green exhibit a homologue in the S. cerevisiae NuA4 complex. The nuclear enrichment score (NES) indicates a nuclear localization if positive. The “ident. in co-IP” column shows in which of the two Co-IPs the protein could be identified. The”identified domains” column displays the domains that were [file ppat.1010514.s001.docx]

| **Co-IP** | **gene ID** | **annotation** | **identified domains** | **Phyre2 modelling** | **NES** | **ident. in Co-IP No.** |
| --- | --- | --- | --- | --- | --- | --- |
| No. 1 | Tb927.11.11530 | histone acetyltransferase HAT2 | SAS2 superfamily | C-terminus: TIP60 (Cov. 67% Conf. 100%) | 5.35 | 1+2 |
| No. 2 | Tb927.11.10070 | Bromodomain, putative, Bdf3 | Bromo-domain | - | 2.3 | 1+2 |
|  | Tb927.3.4140 | hypothetical protein |  | btb domain (Cov. 7%; Conf. 73.3) | 1.55 | 1 |
|  | Tb927.4.2340 | hypothetical protein | - | - bromo-domain from *Leishmania donovani* complexed with bromosporine (Cov. 20%; Conf. 97%);  - crystal structure of tcbdf5 (Cov. 17%; Conf. 97%) | 3.25 | 1+2 |
|  | Tb927.5.3210 | small ubiquitin-related modifier | UBQ/  SUMO | - | 3.05 | 1+2 |
|  | Tb927.6.1070 | hypothetical protein | - | - | 4.82 | 1+2 |
|  | Tb927.7.2770 | hypothetical protein | - | - | 5.81 | 1+2 |
|  | Tb927.9.13320 | hypothetical protein |  | SMAD/FHA domain (Cov. 26%; Conf. 93%) | 2.66 | 1+2 |
|  | Tb927.11.5230 | hypothetical protein | - | ENT-domain of *T. brucei* (Cov. 35%; Conf. 100%) | 2.32 | 1+2 |
|  | Tb927.11.13400 | Bromodomain, putative, Bdf5 | Bromo-domain | - | 3.18 | 1+2 |

**S1A Table Identification of a new complex associated with HAT2**

10 proteins were identified via mass spectrometry in two Co-IP experiments. The initial Co-IP was performed with Tb927.11.11530, the reciprocal Co-IP with the protein Tb927.11.10070 was performed to confirm the Tb927.4.2000 co-IP data. Only Tb927.3.4140 could be identified in the initial but not in the reciprocal Co-IP experiment. The “Annotation” column indicates the curated annotation that was found for the corresponding accession number in the TriTyp database. The” identified domains” column displays the domains that were found by BLAST search using the NCBI database. The Phyre2 modelling column indicates proteins that were identified by homology modelling. Coverage (Cov.) indicates the coverage in percent between query and template. The confidence (Conf.) represents the relative probability in percent (from 0 to 100) that the match between query and template is a true homology. The nuclear enrichment score (NES) indicates a nuclear localization if positive. The last column shows in which of the two Co-IPs the protein could be identified.

| **Co-IP** | **gene ID** | **annotation** | **NES** | **ident. in co-IP No.** | **identified domain** | **Phyre2 modelling** | **Yeast NuA4 subunit** | **Domain(s)** |
| --- | --- | --- | --- | --- | --- | --- | --- | --- |
| No. 2 | Tb927.1.650 | conserved protein, unknown function | 4.38 | 1+2 | - | MORF4 like (Cov. 96% Conf. 95%) | Eaf3/MORF4 | Chromodomain |
|  | Tb927.7.4560 | Histone acetyltransferase 1 | 2.84 | 1+2 | Tudor-knot  Chromo-like domain  MYST HAT | C-terminus: TIP60 (Cov. 66% Conf. 100%)  N-terminus: knotted tudor domain of Esa1 (Cov. 15% Conf. 99%) | Esa1 | Tudor-knot  Chromo-like domain  MYST HAT |
|  | Tb927.7.5310 | YEATS family, putative | 6.59 | 1+2 | YEATS-Domain | Yaf9 /GAS41 (Cov. 14% Conf. 99%) | Yaf9 | YEATS |
| No. 1 | Tb927.9.2910 | histone acetyltransferase subunit NuA4 | 4.59 | 1+2 | Eaf6 | - | Eaf6 | Eaf6 |
|  | Tb927.10.14190 | hypothetical protein, conserved | 5.72 | 1+2 | - | EPL1 (Cov. 63% Conf. 98%) | Epl1 | EpcA |
|  | Tb927.1.3400 | hypothetical protein, conserved | 5.40 | 1+2 | Bromodomain | Bdf5 T.c (Cov. 37% Conf. 97%) |  |  |
|  | Tb927.6.1240 | hypothetical protein, conserved | - | 1 |  |  |  |  |
|  | Tb927.8.5320 | hypothetical protein, conserved | 2.23 | 1+2 |  | - |  |  |
|  | Tb927.10.8310 | histone acetyltransferase 3 | - | 1 | MYST HAT |  |  |  |
|  | Tb927.10.9930 | PHD-zinc-finger like domain | **-** | 1 |  |  |  |  |
|  | Tb927.11.3430 | hypothetical protein, conserved | - | 1+2 | - | - |  |  |

**S1B Table Identification of a new complex associated with HAT1**

11 proteins were identified via mass spectrometry in two co-IP experiments. The initial Co-IP was performed with Tb927.9.2910, the reciprocal Co-IP with the protein Tb927.1.650 was performed to confirm the Tb927.9.2910 co-IP data. Tb927.6.1240, Tb927.10.8310 and Tb927.10.9930 could only be identified in the initial but not in the reciprocal co-IP experiments. The “Annotation” column indicates the curated annotation that was found for the corresponding accession number in the TriTyp database. Proteins labelled in green exhibit a homologue in the *S. cerevisiae* NuA4 complex. The nuclear enrichment score (NES) indicates a nuclear localization if positive. The “ident. in co-IP” column shows in which of the two Co-IPs the protein could be identified. The ”identified domains” column displays the domains that were found by BLAST search using the NCBI / Interpro database. The Phyre2 modelling column indicates proteins that were identified by homology modelling. Coverage (Cov.) indicates the coverage in percent between query and template. The confidence (Conf.) represents the relative probability in percent (from 0 to 100) that the match between query and template is a true homology. The “Yeast NuA4 subunit“ column states the NuA4 complex subunit with its corresponding domain (“domain(s)” column) to which the identified trypanosome protein is homologous to.

| Accession Number | Primer | Sequence |
| --- | --- | --- |
| of the target gene |  |  |
| Tb927.4.2000 | RuvB 2 C-term HA fw | aaaaagactttctcttcggtgaggcagaccgaggggcggagatgtcttacggtgcgcagtgtcagaa***tgaggaagagcag*GGTACCGGGCCCCCCCTCGAG** |
|  | RuvB 2 C-term HA rev | aagctctatatttatcttttttctttttatgtgctgccattctactcacgccaccctctttccctggagaaaaggaaaca**TGGCGGCCGCTCTAGAACTAGTGGAT** |
|  | RuvB 2 int pcr fw | **CATATTTGTTTCCCCCTACACG** |
|  | RuvB 2 int pcr re | **CTTTTTATGTGCTGCCATTCTAC** |
|  |  |  |
| Tb927.10.11690 | TbSWRC1 RNAi fw | ggggacaagtttgtacaaaaaagcaggct**GAAGAAGCTGAAGATCAGCT** |
|  | TbSWRC1 RNAi rev | ggggaccactttgtacaagaaagctgggt**GAAAGCCGTTAGGAAGAGT** |
|  | TbSWRC1 int PCR fw | **GTGCTGGAATAGAGGGAGAA** |
|  | TbSWRC1 int PCR rev | **CAACATCCTAAACCTGTAACCA** |
|  | TbSWRC1 HA N-term fw | tgcggtgctgtgatagtggggaaccataggggcaaggtgctggaatagagggagaacgtgtgtggtgtagttgaagttca**GTATAATGCAGACCTGCTGC** |
|  | TbSWRC1 HA N-term rev | gtggcacctgtgtaaactacatattcgtgttcccccgtgatggaaacagaagatccagtagatttagaagtggcctgcat***ctcgagggggggcccggtacc***CGCGTAATCTGGCACGTC |
|  | TbSWRC1 neo KO fw | tgcggtgctgtgatagtggggaaccataggggcaaggtgctggaatagagggagaacgtgtgtggtgtagttgaagttca**ATGATTGAACAAGATGGATTGCA** |
|  | TbSWRC1 neo KO rev | acggtaaatgccgctcgcttggttaacgcccttcaacatcctaaacctgtaaccatgaccacacgcaaggaacacactcc**TCAGAAGAACTCGTCAAGAAGG** |
|  |  |  |
| Tb927.11.5830 | TbSWRC2 RNAi fw | ggggacaagtttgtacaaaaaagcaggct**TCAGTACTGGTCGTGCACAC** |
|  | TbSWRC2 RNAi rev | ggggaccactttgtacaagaaagctgggt**CCGTTTCTTTGCAGCTGCTT** |
|  | TbSWRC2 int PCR fw | **CATTACCGTGTAGCCTTTCAG** |
|  | TbSWRC2 int PCR rev | **CATGTTGATAATGCGCCAGC** |
|  | TbSWRC2 HA N-term fw | caaaatttatctaccatttctcacgctagttacttcattaccgtgtagcctttcagtactggtcgtgcacacttccaact**GTATAATGCAGACCTGCTGC** |
|  | TbSWRC2 HA N-term rev | ttttggagcatattgccacggttggcacgccgcgggcggtcgtcagcgacattccagcgggagtcgtcaacaccgtccat***ctcgagggggggcccggta*ccCGCGTAATCTGGCACGTC** |
|  | TbSWRC2 neo KO fw | caaaatttatctaccatttctcacgctagttacttcattaccgtgtagcctttcagtactggtcgtgcacacttccaact**ATGATTGAACAAGATGGATTGCA** |
|  | TbSWRC2 neo KO rev | cagcctccagatcgatgtgaatgcaccctctgtcaacgatctgtgttacactacacgaaacatcatccaacatcacccga**TCAGAAGAACTCGTCAAGAAGG** |
|  |  |  |
| Tb927.7.4040 | TbSWRC4 C-term HA fw | tgcgcaggtttgctgggaggtgtggtactctcaaggcctcacttaaaaaacttgcgagtgacgcttcttcaatgaaaata**GGTACCGGGCCCCCCCTCGAG** |
|  | TbSWRC4 C-term HA rev | cgacgatagtgacaatatgtgccagacttgagagatgctggccaacaaatgggtaaataggagtggttgagcagcaatcc**TGGCGGCCGCTCTAGAACTAGTGGAT** |
|  | TbSWRC4 int PCR fw | **GGATGAATGTGTAAGTAGGTTGC** |
|  | TbSWRC4 int PCR rev | **GTGCCAGACTTGAGAGATG** |
| Tb927.11.11530 | HAT2 HA N-term fw | cccaccggcaacatagctgtagcggttttgtgatcggaggttgagagttctgttgcctagtggatgcgagaggttgcgca**GTATAATGCAGACCTGCTGC** |
|  | HAT2 HA N-term rev | AAGAGTTGTTGGAGCTGATCCTCTTCCTCAGACGGGCCCCCATTGATGTCACCTCCGTCTTTTTTTGCTGCTAACGACGC  ***ctcgagggggggcccggtacc*CGCGTAATCTGGCACGTC** |
|  | HAT2 int PCR fw | **CCGCCTTCATTCATTTCTCC** |
|  | HAT2 int PCR rev | **GAGTAAGACAGAAACCGCAGA** |
|  | HAT2 neo KO fw | cccaccggcaacatagctgtagcggttttgtgatcggaggttgagagttctgttgcctagtggatgcgagaggttgcgca**ATGATTGAACAAGATGGATTGCA** |
|  | HAT2 neo KO rev | acagaaaccgcagaagcactgactctgggaaaggtaagttagaaataaaagaccatttatattgtgggagggacgacaca**TCAGAAGAACTCGTCAAGAAGG** |
|  | HAT2 RNAi fw | ggggacaagtttgtacaaaaaagcaggct**CCCACCACAGTGCGATACTT** |
|  | HAT2 RNAi rev | ggggaccactttgtacaagaaagctgggt**AAGTGACGATGCATGCCAGA** |
|  |  |  |
| Tb927.11.10070 | Bdf3 TY N-term fw | gagagcaaaaaaagaaattgtgttaccgaagtcacacattgagaaaacctagtggcaaccagtaagtattgcgcgcatcg**CGTATAATGCAGACCTGCTGC** |
|  | Bdf3 TY N-term rev | agtggatggctgagtgcgcagtcgatgtacctgatccaatagtttatatcttcagcaagtttgacccctgtgaggaccat**CCTTGGGTCAAGTGGGTC** |
|  | Bdf3 int PCR fw | **CAAGTATACCAACAAGCCCG** |
|  | Bdf3 int PCR rev | **AACCATGCCGCTGTCTACA** |
|  | Bdf3 RNAi fw | ggggacaagtttgtacaaaaaagcaggct**CACTCAGCCATCCACTACCG** |
|  | Bdf3 RNAi rev | ggggaccactttgtacaagaaagctgggt**TGAGTGTTGGGTCTTCACGG** |
|  |  |  |
| Tb927.4.5020 / | RNA Pol II RNAi fw | ggggacaagtttgtacaaaaaagcaggct**CGAAGGAGCTGACTCGATCC** |
| Tb927.8.7400 | RNA Pol II RNAi rev | ggggaccactttgtacaagaaagctgggt**GACGTATCGGAGCGGTTGAT** |
|  |  |  |
|  | Luci pFG14 fw | gactattcatccgtttatattagcaacagtaggtactagcaccactaacaacaacaacaaagcacttctatttatttatc**ATGATTGAACAAGATGGATTGCA** |
|  | Luci pFG14 rev | gagacagaaaacaaattcacaaaagaagaaagaaagaacataaatgaaaacctacacatggtgcgacgttgtcacacttt**TTATTGTTCATTTTTGAGAACTCGC** |
|  |  |  |
|  | pPOT HA insert HindIII fw | cagatcaagctt**ATGTACCCTTACGATGTGCCT** |
|  | pPOT HA insert NheI BamHI rev | *atgactggatcccgcgtaatctggcacgtc*gtacgggtaagcatagtccggaacatcgtatggata**TGCGTAATCGGGCACATC** |
|  | pPOT TY insert HindIII fw | ggttctaagctt**ATGGAGGTCCATACTAACCAGGAC** |
|  | pPOT TY insert BamHI rev | *cagtcggatcct*tcaagaggtggtcctgtaccgtcaagtgggtcctggttagtatggacctcccttg**GGTCAAGTGGGTCCTGGTTAG** |
|  | pPOTv7 hyg EcoRI fw | *gactgaattc***ATGAAAAAGCCTGAACTCACC** |
|  | pPOTv7 hyg NcoI rev | *gactaccatgg***TTATTCCTTTGCCCTCGG** |
|  | pPOTv7 seqeuncing fw | **CAAGTATACCAACAAGCCCG** |
|  |  |  |
|  | Neo pMO Hind III fw | *gtcagctagcttaagcttgggctagaactagtg***ATGATTGAACAAGATGGATTGCA** |
|  | Neo pMO BamHI rev | *tactggatccgcgttcggtcggcatctact***TCAGAAGAACTCGTCAAGAAGG** |
|  | Blas pMO HindIII fw | *gtcagctagcttaagcttgggctagaactagtg***ATGCCTTTGTCTCAAGAAGAATC** |
|  | Blas pMO BamHI rev | *tactggatccgcgttcggtcggcatctact***TTAGCCCTCCCACACATAAC** |
|  | Hyg pMO Hind III fw | *gtcagctagcttaagcttgggctagaactagtg***ATGAAAAAGCCTGAACTCACC** |
|  | Hyg pMO BamHI rev | *tactggatccgcgttcggtcggcatctact***TTATTCCTTTGCCCTCGG** |
|  |  |  |
|  |  |  |
|  | Primer code: | **Bold**: primer binding site |
|  |  | ***italic bold:*** linker |
|  |  | underlined: sequence coding for part of the tag |
|  |  | normal: recombination sequences |
|  |  | *italic*: sequences that contain one or more restriction sites |

**S1C Table Primer list**

Primer sequences that were used for cloning of RNAi and knock-out constructs and PCR amplification for *in situ* tagging
