# Supplementary material for: A novel SNF2 ATPase complex in Trypanosoma brucei with a role in H2A.Z-mediated chromatin remodelling
Source: PLoS Pathog. 2022 Jun 8;18(6):e1010514. doi: 10.1371/journal.ppat.1010514 (PMC9236257; doi:10.1371/journal.ppat.1010514)
Supplement: S1 Fig — A Fig Trypanosoma brucei possesses a SNF2 protein with characteristics of the SWR1 subfamily. A database search for SNF2 ATPases in T. brucei identified 15 proteins that putatively belong to the SNF2 superfamily. Only the protein Tb927.11.10730 has the characteristic structure of a protein of the SWR1 subfamily. For comparison, the structure of the SWR1 protein from S. cerevisiae is depicted (S.c. SWR1). In addition to the DEXQ DEAD-Box motif, a key feature of the SWR1 members of the SNF2 superfamily is an insertion (red box) between the DEAD-box and the helicase C domain. B Fig Summary of volcano plots overview of 4 co-IPs. Volcano blot of co-purified proteins after (A) WT control vs. HA-TbSWRC1 (Tb927.10.11690), (B) WT control vs. HA-TbSWRC2 (Tb927.11.5830), (C) WT control vs. TbSWRC4-HA (Tb927.7.4040) and (D) WT control vs. HA-RuvB2 (Tb927.4.2000), co-IPs obtained by MS analysis of four biological replicates. Green dots represent purified proteins with a p-value of > 0.01 or with a fold-enrichment of = / > 1. Blue dots represent purified proteins with a p-value = / < 0.01 or with a fold-enrichment of > 1. Orange dots represent the proteins with a p-value = / < 0.01 or with a fold-enrichment of > 1 that could be identified in at least three of the four co-IPs. The annotations “measured” indicates that a sufficient number of unique peptides of the protein could be detected in the control samples to identify the corresponding protein. The annotation “some imputed” or “imputed” indicate that a theoretical value had to be imputed for some unique peptides that were used to identify the protein. C Fig Depletion of TbSWR1 reduces the amount of chromatin associated H2A.Z. (A) Growth of parasites was monitored for 96 hours after RNAi-mediated depletion of TbSWR1 (Tb927.11.10730) using tetraycline (tet). The parental 2T1 cell line was used as a control (n = 3). (B) Quantification of live/dead staining with propidium iodide of TbSWR1-depleted cells at the indicated timepoint [file ppat.1010514.s003.docx]

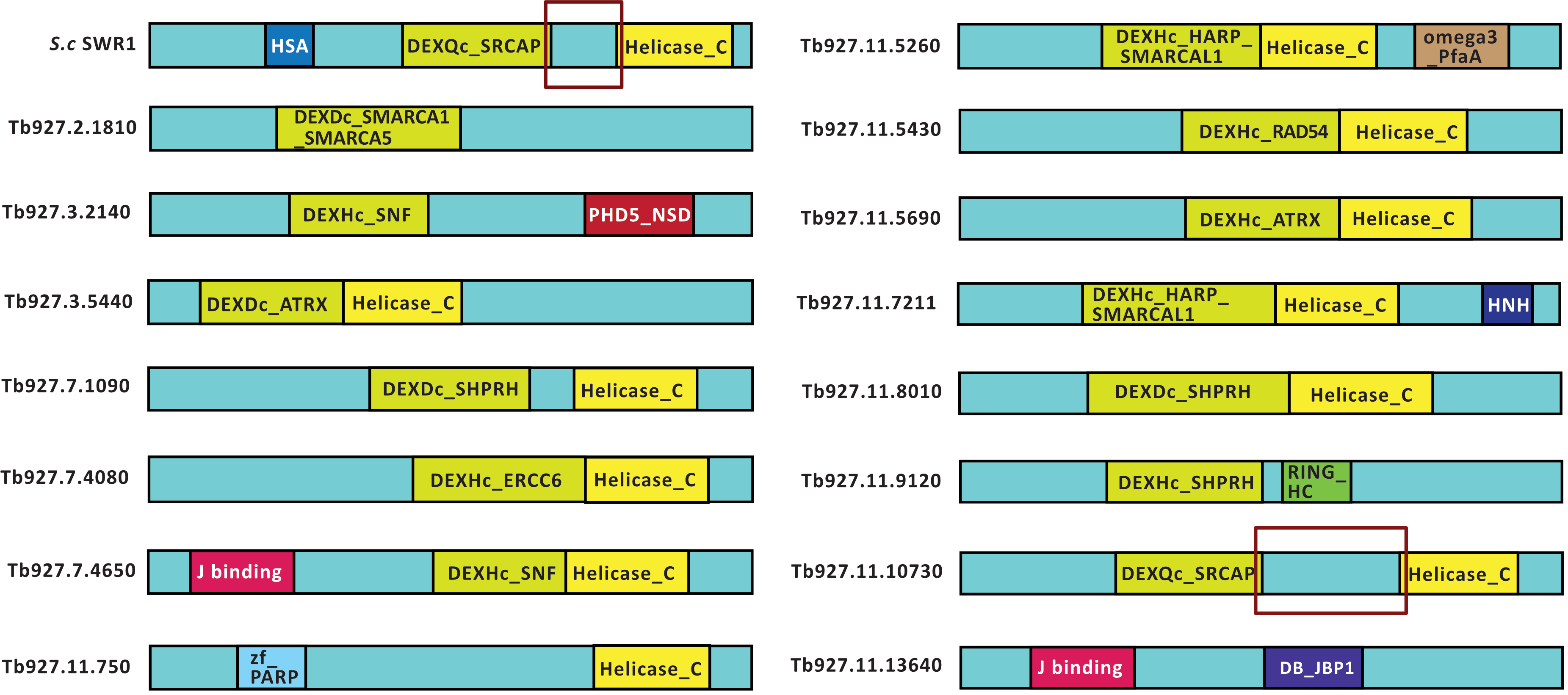


**S1A Fig *Trypanosoma brucei* possesses a SNF2 protein with characteristics of the SWR1 subfamily**

A database search for SNF2 ATPases in *T. brucei* identified 15 proteins that putatively belong to the SNF2 superfamily. Only the protein Tb927.11.10730 has the characteristic structure of a protein of the SWR1 subfamily. For comparison, the structure of the SWR1 protein from *S. cerevisiae* is depicted (*S.c.* SWR1). In addition to the DEXQ DEAD-Box motif, a key feature of the SWR1 members of the SNF2 superfamily is an insertion (red box) between the DEAD-box and the helicase C domain.

**
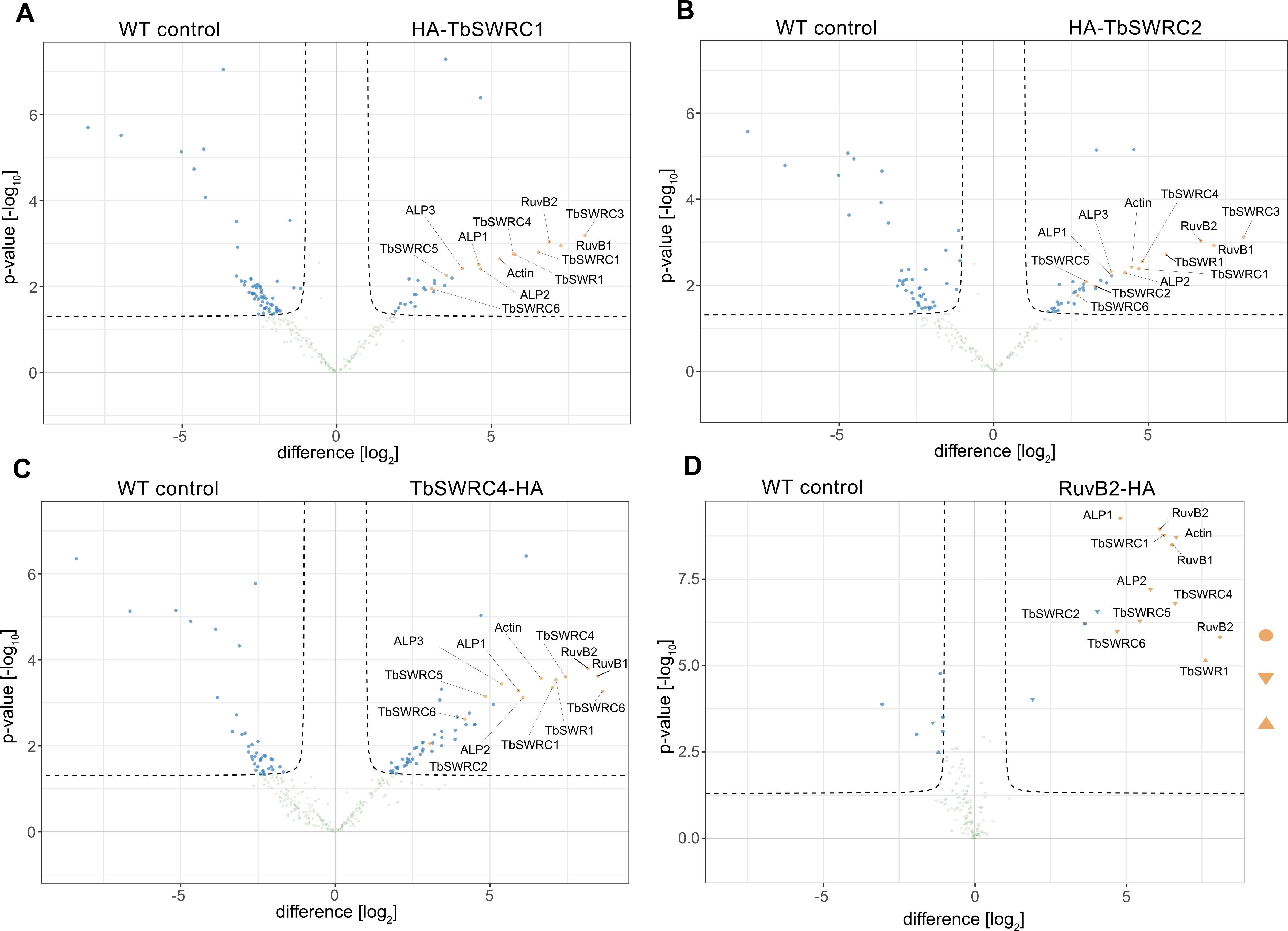
**

imputed

some

imputed

measured

**S1B Fig Summary of volcano plots overview of 4 co-IPs**

Volcano blot of co-purified proteins after **(A)** WT control vs. HA-*Tb*SWRC1 (Tb927.10.11690), **(B)** WT control vs. HA-*Tb*SWRC2 (Tb927.11.5830), **(C)** WT control vs. *Tb*SWRC4-HA (Tb927.7.4040) and **(D)** WT control vs. HA-RuvB2 (Tb927.4.2000), co-IPs obtained by MS analysis of four biological replicates. Green dots represent purified proteins with a p-value of > 0.01 or with a fold-enrichment of = / > 1. Blue dots represent purified proteins with a p-value = / < 0.01 or with a fold-enrichment of > 1. Orange dots represent the proteins with a p-value = / < 0.01 or with a fold-enrichment of > 1 that could be identified in at least three of the four co-IPs. The annotations “measured” indicates that a sufficient number of unique peptides of the protein could be detected in the control samples to identify the corresponding protein. The annotation “some imputed” or “imputed” indicate that a theoretical value had to be imputed for some unique peptides that were used to identify the protein.


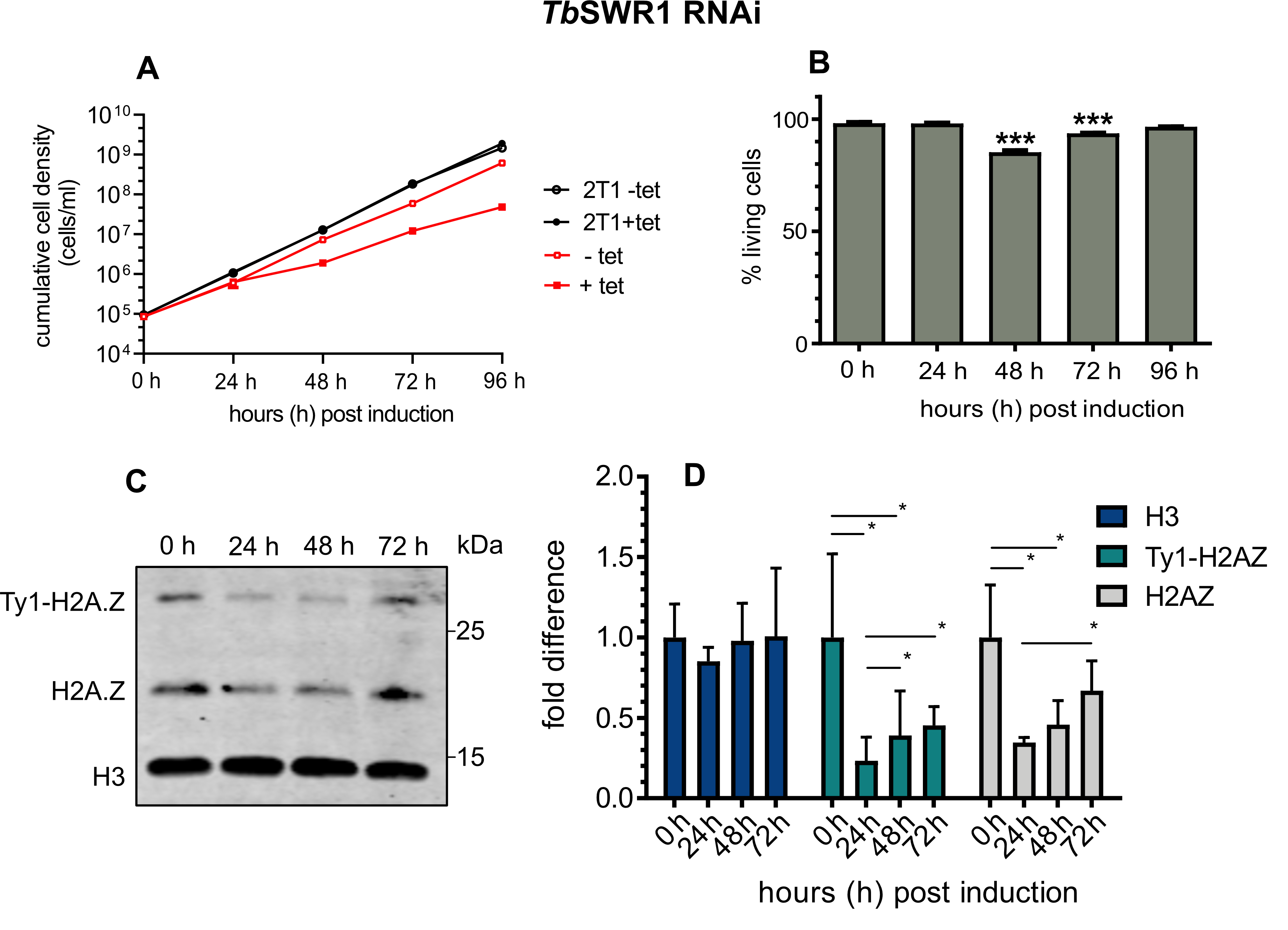


**S1C Fig Depletion of *Tb*SWR1 reduces the amount of chromatin associated H2A.Z**

**(A)** Growth of parasites was monitored for 96 hours after RNAi-mediated depletion of *Tb*SWR1 (Tb927.11.10730) using tetraycline (tet). The parental 2T1 cell line was used as a control (n=3). **(B)** Quantification of live/dead staining with propidium iodide of *Tb*SWR1-depleted cells at the indicated timepoints post-induction. Analysis was done by flow cytometry (n=3). **(C)** Western blot analysis of the insoluble nuclear fraction with antibodies specific for histone H3 and the histone variant H2A.Z. Lysates from an equal number of cells (2x10^6^ per lane) were analysed for each timepoint. **(D)** Quantification of chromatin-associated H3 (dark blue), Ty1-H2A.Z (turquoise) and H2AZ (grey) (N=3 for all depicted experiments; *** = p-value <0.001; ** = p‑value 0.001-0.01; * = p‑value 0.01-0.05).

**
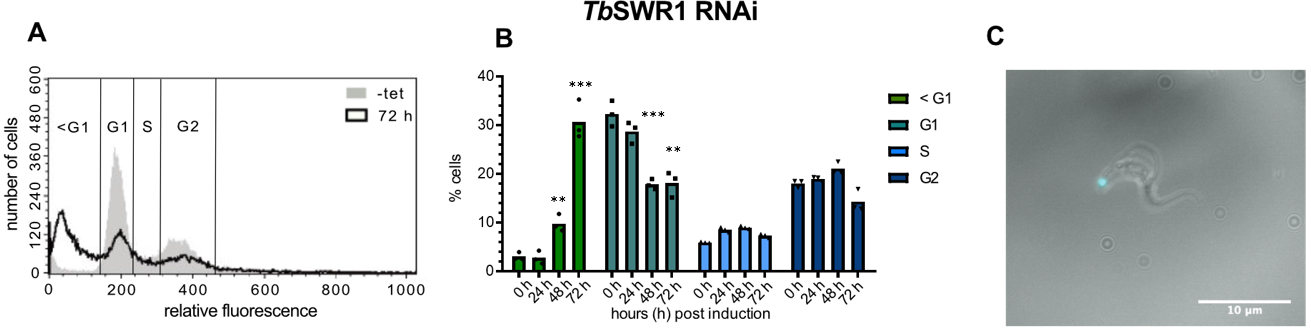
**

**S1D Fig Depletion of *Tb*SWR1 leads to anucleated cell**

**(A)** Exemplary cell cycle profile of bloodstream form cells without (grey line) *Tb*SWR1 (Tb927.11.10730) depletion and after 72 h of protein depletion (black line). The Gates show the different populations of sub G1-, G1-, S- and G2-Phase cells. **(B)** Data of three triplicates, sub G1 Phase cells (green), G1-Phase cells (green‑blue), S-Phase cells (light blue) and G2-Phase cells (dark blue). The data show a decrease of cells in G1 and G2 Phase in addition to the increase of sub G1‑Phase cells (n=3 for all depicted experiments; *** = p-value <0.001; ** = p‑value 0.001-0.01; * = p‑value 0.01-0.05). **(C)** Light microscopy images (N=1) of a BSF cell after 72h of *Tb*SWR1 depletion. Scale bar 10µm.

**
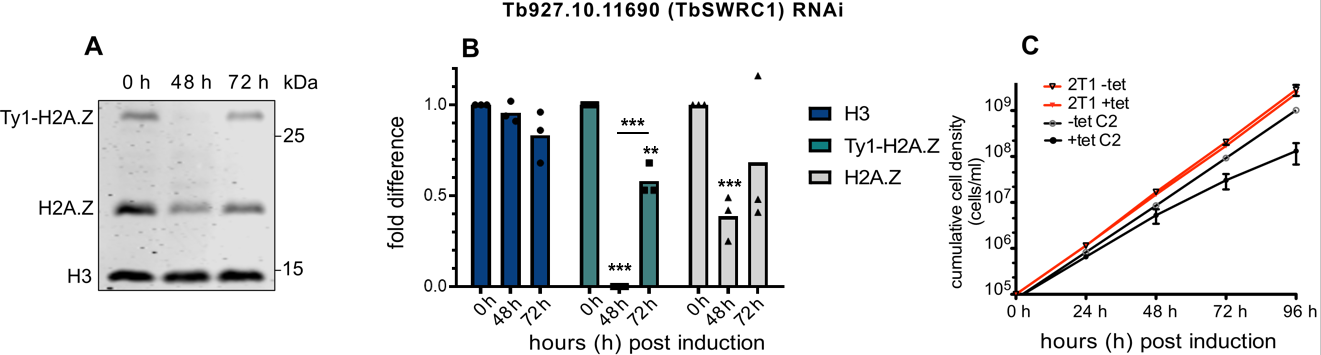
**

**S1E Fig Depletion of *Tb*SWRC1 (Tb927.10.11690) reduces the amount of chromatin associated H2A.Z**

**(A)** Exemplary Western Blot analysis of the nuclear fraction with antibodies against histone H3 and the histone variant H2A.Z. An equal amount of cell equivalent was loaded for each timepoint. **(B)** The development of chromatin associated H3 (dark blue), Ty1-H2A.Z (turquoise) and H2A.Z (grey) in course of *Tb*SWRC1 depletion is plotted (N=3). **(C)**Growth of parasites was monitored for 96 hours after RNAi-mediated depletion of *Tb*SWRC1 using tetracycline (tet). Growth of tet induced and non-induced parental 2T1 cells was measured for 96h and acts as a reference (N=3 for all depicted experiments; *** = p-value <0.001; ** = p‑value 0.001-0.01; * = p‑value 0.01-0.05).

**
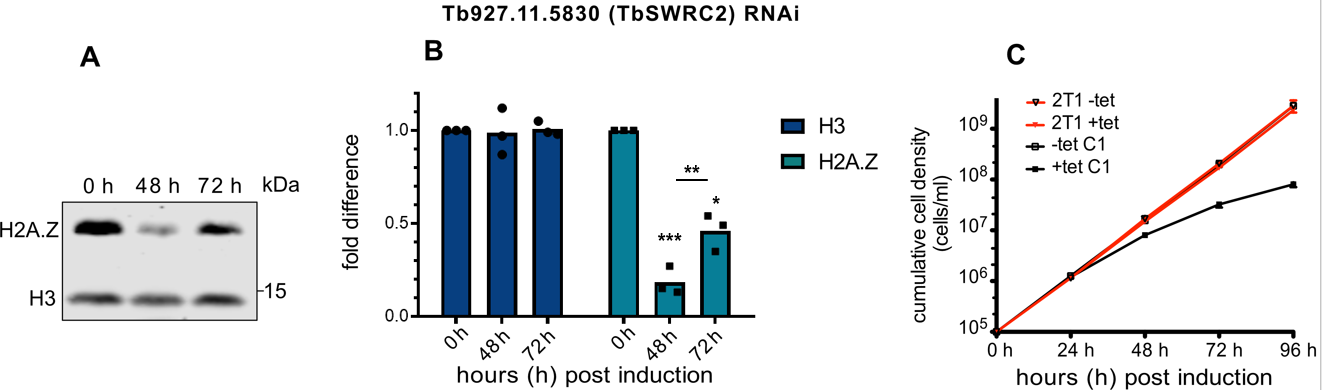
**

**S1F Fig Depletion of *Tb*SWRC2 (Tb927.11.5830) reduces the amount of chromatin associated H2A.Z**

**(A)** Exemplary Western Blot analysis of the nuclear fraction with antibodies against histone H3 and the histone variant H2A.Z. An equal amount of cell equivalent was loaded for each timepoint. **(B)** The development of chromatin associated H3 (dark blue) and H2A.Z (turquoise) in course of *Tb*SWRC2 depletion is plotted (N=3). **(C)** Growth of parasites was monitored for 96 hours after RNAi-mediated depletion of *Tb*SWRC2 using tetracycline (tet). Growth of tet induced and non-induced parental 2T1 cells was measured for 96h and acts as a reference (N=3 for all depicted experiments; *** = p-value <0.001; ** = p‑value 0.001-0.01; * = p‑value 0.01-0.05).

**
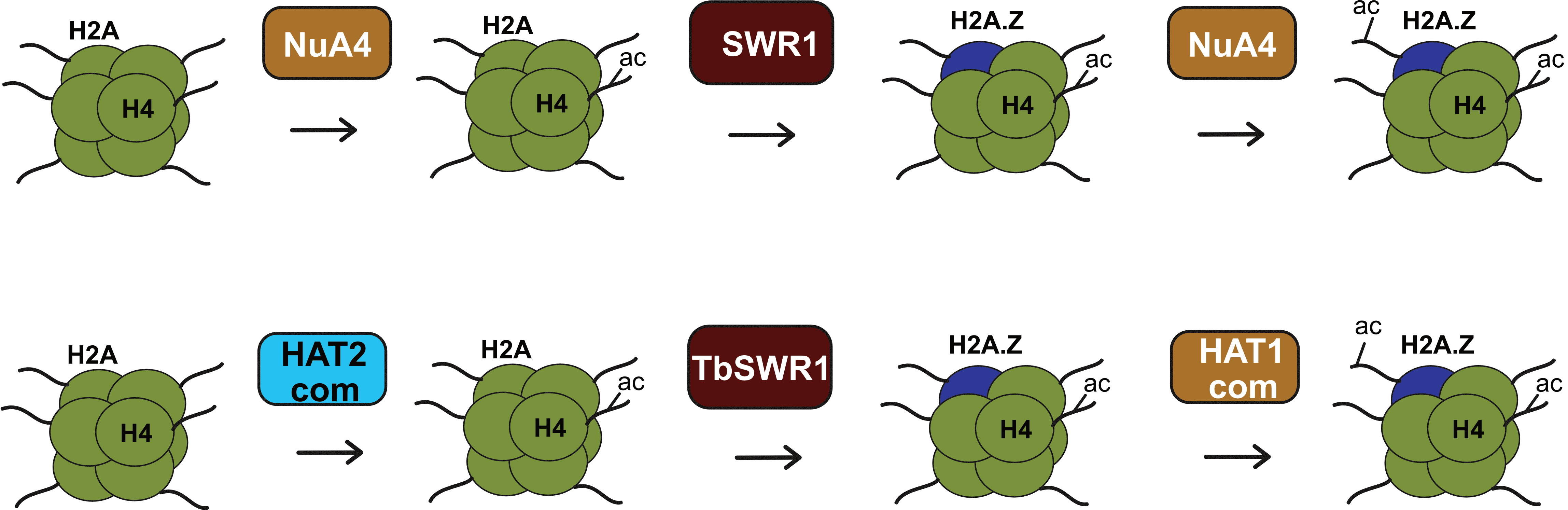
**

**S1G Fig H2A.Z acetylation pathway in *S. cerevisiae* and *T. brucei***

Depiction of the H2A.Z acetylation pathway in *S. cerevisiae* (top panel) and *T. brucei* (bottom panel). In *S. cerevisiae* the NuA4 complex acetylates histone H4 to facilitate SWR1 recruitment to the nucleosome. SWR1 exchanges H2A with H2A.Z (10, 29, 30, 58). Subsequent to the exchange the NuA4 complex acetylates H2A.Z which enhances transcription. In *T.brucei* two distinct HAT-complexes are responsible for acetylation of histone H4 and histone H2A.Z. While H4 is the substrate for the HAT2 complex, H2A.Z is acetylated by the HAT1 complex.

**
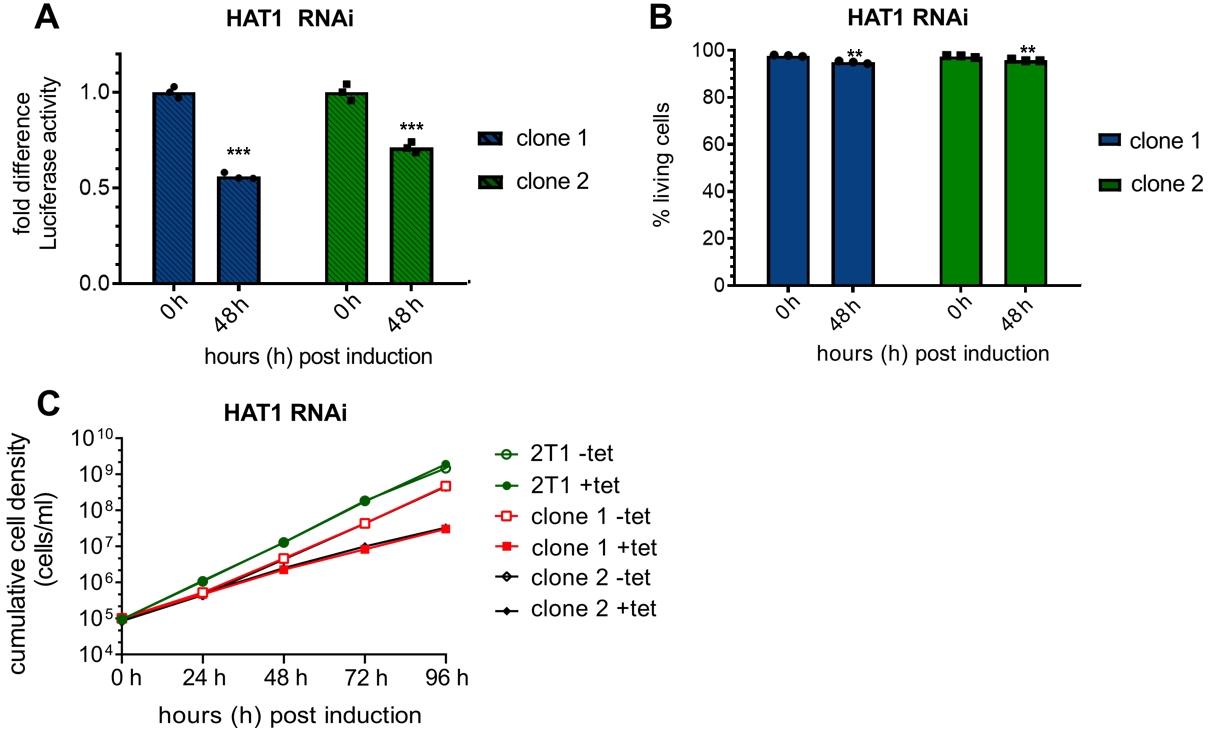
**

**S1H Fig Depletion of the histone acetyltransferase HAT1 caused a decrease of reporter luciferase activity within a PTU**

A single luciferase reporter construct was integrated into the tubulin array of a HAT1 (Tb927.7.4560) RNAi cell line. Samples for the luciferase assay were normalised to cell numbers. **(A)** Luciferase activity was monitored for 48 h after induction of RNAi in two independent clones. Values of non-induced cells were set to 1. **(B)** Live/dead staining of each RNAi cell line was performed in triplicates at the same time points. **(C)** Growth of parasites was monitored for 96 hours after RNAi-mediated depletion of H2A.Z using tetracycline (tet) induction. Growth of the parental 2T1 cell line was measured for 96h as a control. (N=3 for all depicted experiments; *** = p-value <0.001; ** = p‑value 0.001-0.01; * = p‑value 0.01-0.05).

**
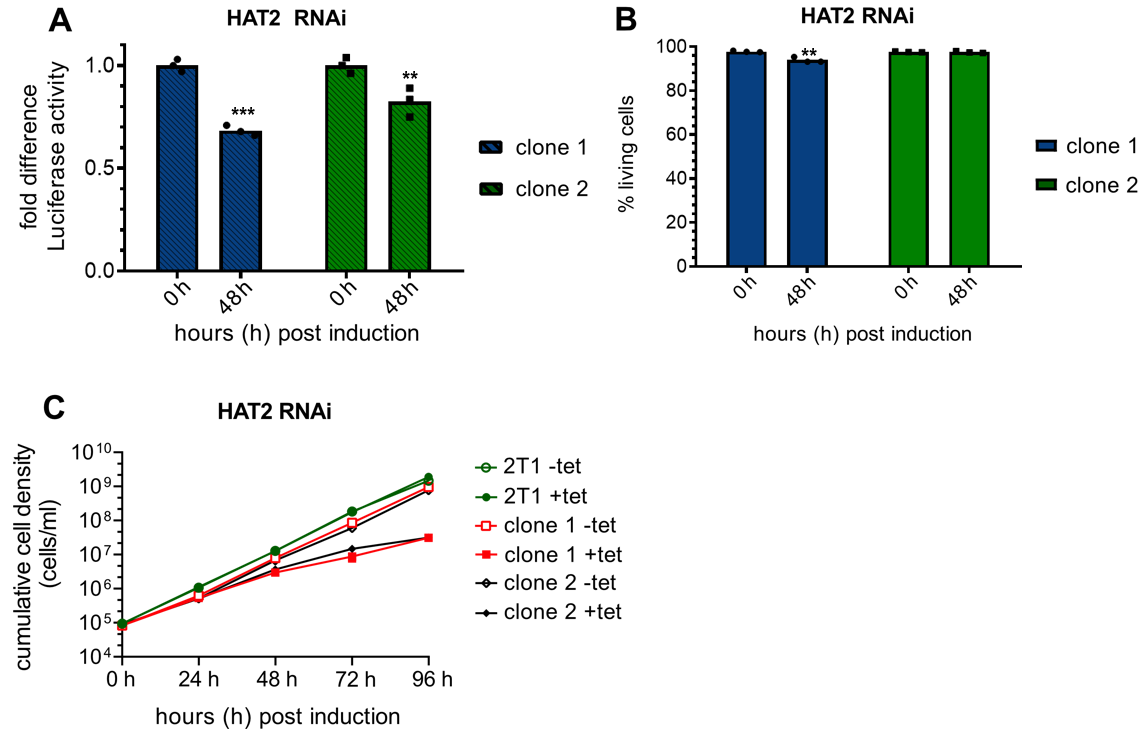
**

**S1I Fig Depletion of the histone acetyltransferase HAT1 caused a decrease of reporter luciferase activity within a PTU**

A single luciferase reporter construct was integrated into the tubulin array of a HAT2 (Tb927.11.11530) RNAi cell line. Samples for the luciferase assay were normalised to cell numbers. **(A)** Luciferase activity was monitored for 48 h after induction of RNAi in two independent clones. Values of non-induced cells were set to 1. **(B)** Live/dead staining of each RNAi cell line was performed in triplicates at the same time points. **(C)** Growth of parasites was monitored for 96 hours after RNAi-mediated depletion of H2A.Z using tetracycline (tet) induction. Growth of the parental 2T1 cell line was measured for 96h as a control. (N=3 for all depicted experiments; *** = p-value <0.001; ** = p‑value 0.001-0.01; * = p‑value 0.01-0.05).

**
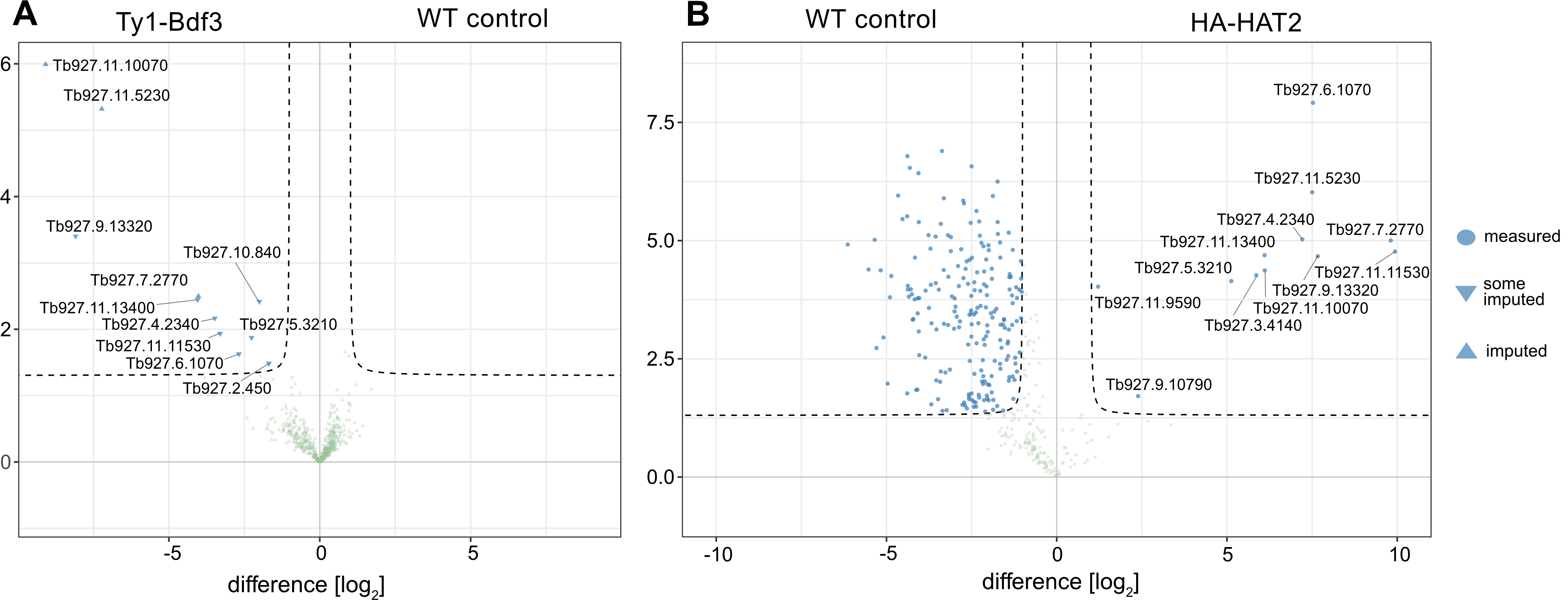
**

**S1J Fig Identification of a HAT2 complex**

Volcano blot of co-purified proteins after **(A)** Ty1-Bdf3 (Tb927.11.10070) vs. WT control **(B)** WT control vs. HA-HAT2 (Tb927.11.11530), co-IPs obtained by MS analysis of four biological replicates. Green dots represent purified proteins with a p-value of > 0.01 or with a fold-enrichment of = / > 1. Blue dots represent purified proteins with a p-value = / < 0.01 or with a fold-enrichment of > 1. The annotations “measured” indicates that a sufficient number of unique peptides of the protein could be detected in the control samples to identify the corresponding protein. The annotation “some imputed” or “imputed” indicate that a theoretical value had to be imputed for some unique peptides that were used to identify the protein.

**
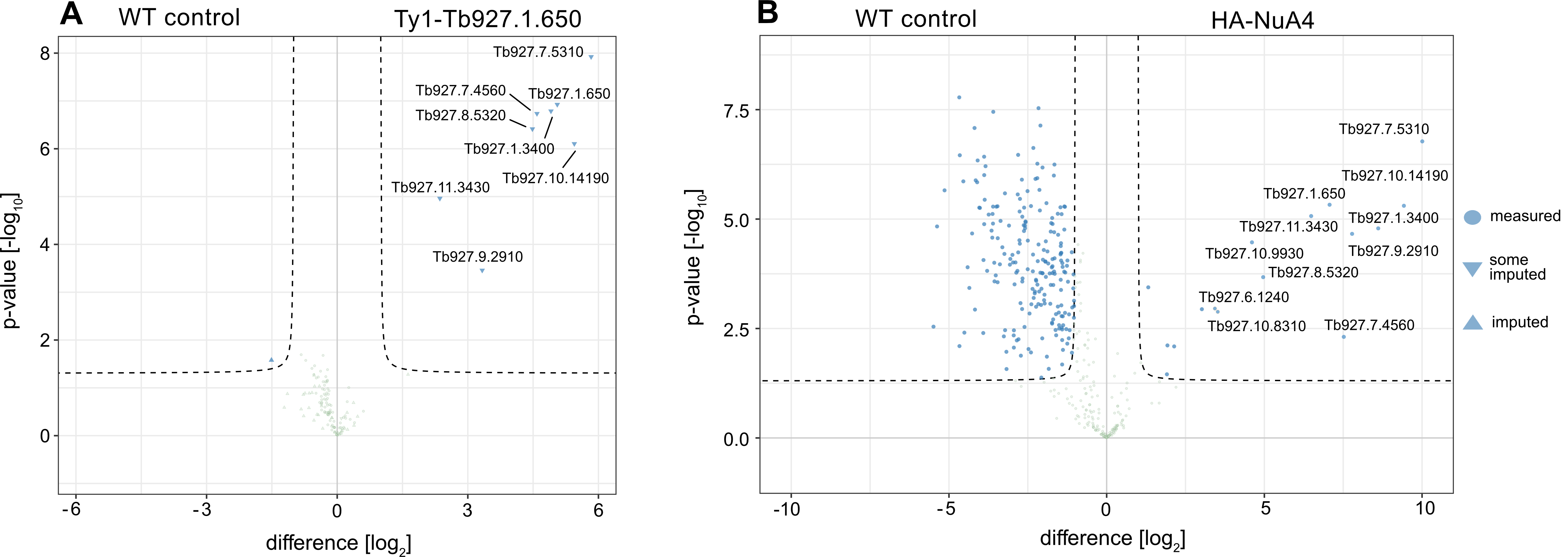
**

**S1K Fig Identification of a HAT1 complex**

Volcano blot of co-purified proteins after **(A)** WT control vs. Ty1-Bdf3 (Tb927.1.650) **(B)** WT control vs. HA-HAT2 (Tb927.9.2910), co-IPs obtained by MS analysis of four biological replicates. Green dots represent purified proteins with a p-value of > 0.01 or with a fold-enrichment of = / > 1. Blue dots represent purified proteins with a p-value = / < 0.01 or with a fold-enrichment of > 1. The annotations “measured” indicates that a sufficient number of unique peptides of the protein could be detected in the control samples to identify the corresponding protein. The annotation “some imputed” or “imputed” indicate that a theoretical value had to be imputed for some unique peptides that were used to identify the protein.


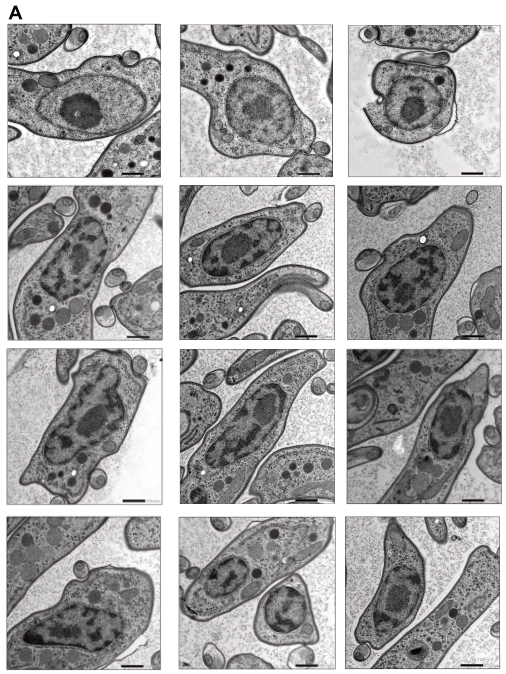


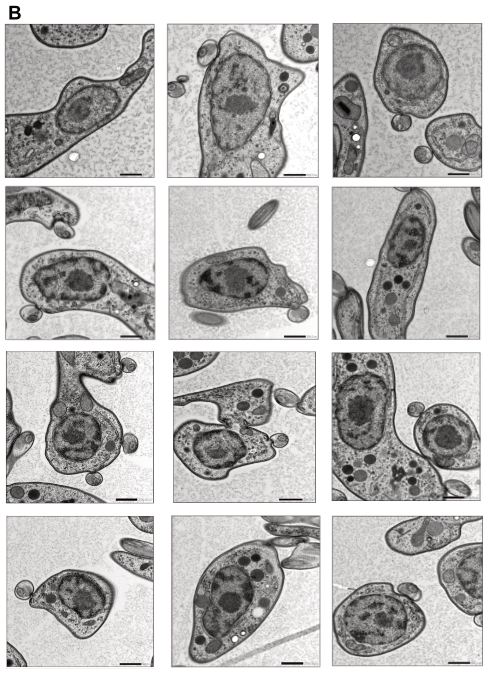


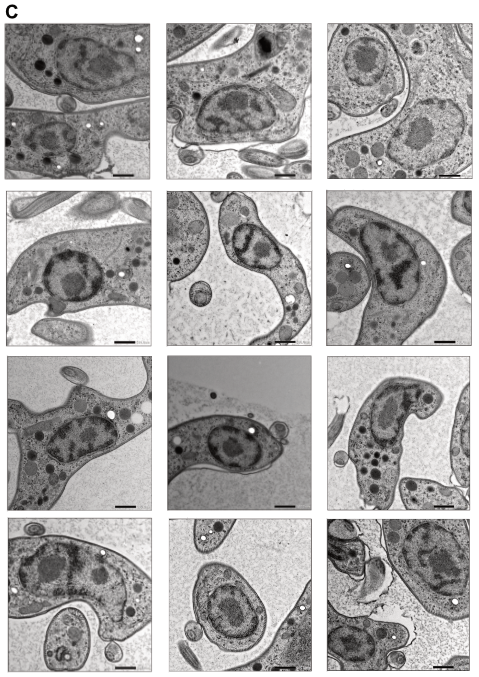


**S1L Fig Chromatin condensation after *Tb*SWR1, H2A.Z and RNAP II (RPB1) depletion**

Representative electron microscopy images of the nucleus of *Tb*SWR1 **(A)**, H2A.Z **(B)** and RNAP II (RPB1; **C)** depleted cells. The upper panel depicts uninduced cell lines, the three panels below depicts TET induced cells (*Tb*SWR1 and H2A.Z: 24 h RNAi, RPB1: 16h RNAi). Depletion of the proteins resulted in large black patches of condensed chromatin. Scale bar, corresponds to 500 nm. Images obtained using a STEM (scanning transmission electron microscope).

**S1M Fig: Reduction in SWR1 mRNA molecules following RNAi depletion**

The number of SWR1 mRNA molecules per cell was measured by single molecule FISH. in the absence of TET and after 24 hours RNAi induction. Several unrelated mRNAs served as controls, as indicated. Affymetrix probe sets were designed antisense to the full ORF (*BDF3*), full ORF (*ZFP1*), 1100 most 5´nucleotides (Tb427.01.1730), full ORF (*CAF1*) and the repetitive sequence of *FUTSCH*. At least 46 cells were counted for each probe and timepoint. The data are presented as box-plots (waist is median; box is interquartile range (IQR); whiskers are 1.5 IQR). The differences in mRNA numbers in the absence and presence of SWR1 RNAi were evaluated by a students t-TEST (two samples, two-tailed; * for <0.05 = significant; *** for <0.005 = highly significant). There was a highly significant reduction in the number of SWR1 mRNA molecules upon RNAi induction. Note that two of the four control mRNAs also showed a significant reduction in numbers: this is expected from the general reduction in transcription that is a consequence of SWR1 depletion.
