# Supplementary material for: A novel SNF2 ATPase complex in Trypanosoma brucei with a role in H2A.Z-mediated chromatin remodelling
Source: PLoS Pathog. 2022 Jun 8;18(6):e1010514. doi: 10.1371/journal.ppat.1010514 (PMC9236257; doi:10.1371/journal.ppat.1010514)
Supplement: Attachment — Submitted filename: response to reviewer.pdf [file ppat.1010514.s004.pdf]

Wuerzburg, September 7th 2021

Dear Editor

Thank you for your letter and the referee's comments on our manuscript. We were happy to learn that the referees' comments on our manuscript were so positive. We have addressed all of the reviewer's comments below in details. We believe this manuscript improved due to the reviewer's suggestions and hope that you might be willing to consider the revised manuscript for publication in *PLoS Pathogen*.

Sincerely,

Falk Butter & Christian Janzen

Reviewer's Responses to Questions

Part I – Summary

Reviewer #1: In this paper the authors have identified trypanosome chromatin remodelling complexes in the T. brucei by mass spectrometry, and shown that depletion of a component leads to a decrease in steady-state mRNA and deposition of histone H2AZ.

Reviewer #2: The chromatin remodeling complex SWR1 and its involvement in replacing canonical histone 2A in nucleosomes with the variant H2A.Z is conserved across the eukaryotic kingdom. Since H2A.Z is also found in Trypanosoma brucei, being specifically enriched in transcription initiation regions, Vellmer et al. set out to characterize trypanosome components of the SWR1 remodeling complex. Using co-IP experiments, they identified several conserved and putative SWR1 subunit homologs which might form a complex in trypanosomes (and the same is true for the HAT2 co-purified proteins). However, there is no additional evidence (second criterion) that these proteins indeed form the complex envisioned by the authors. The complex drawn in Fig. 1A is at best a model of a putative trypanosome complex and the work an initial characterization of SWR1 complex homologs, not more. The title therefore is an overstatement and even more so the statement (lines 195/6): "The 13 proteins that were identified in at least three of the four co-IP experiments assemble the trypanosome SWR1-like complex". There is no assembly assay whatsoever.
While the authors present convincing data that trypanosome SWR1, as its eukaryotic counterparts, is essential for H2A.Z abundance and deposition, the study is mainly confirmatory in nature in this respect. Identifying SWR1 in the genome database should have been straightforward since it is by far the most conserved homolog to yeast SWR1 (E=e-99 versus e-64 to the next potential homolog). Moreover, the knockdowns of the study were restricted to clear homologs of yeast proteins and did not include putative trypanosome-specific genes, making their impact on H2A.Z predictable. Hence, this work provides only moderate progress in understanding trypanosomatid-specific SWR1 complex features. In addition, there are several concerns listed below.

Reviewer #3: This manuscript describes a series of co-immunoprecipitation experiments that identify 13 proteins forming an SWR1-like chromatin remodeling complex in Trypanosoma brucei and a serious of RNAi experiments that show it plays a critical role in H2A.Z incorporation at transcription start sites. With a few exception 9some of which are noted below), the manuscript is well-written, and the results will be of interest to most readers. However, a few (relatively minor) issues need to be addressed.

Part II – Major Issues: Key Experiments Required for Acceptance

Reviewer #1:

1. Deposit the proteomics and sequence data in a public database and give the accession numbers
Access to the MS proteomics data and the ChIPseq metadata have been added to the material and methods section.
2. Provide an Excel Table as a supplement for the quantitative MS.
The information was added as supplementary material S4.
3. All new gene assignments must be annotated using user comments in TritrypDB.
Our plan is to do this once the manuscript passed the peer review process.
4. When there are only three measurements (which is everywhere) please replace bar graphs and

standard deviations with plots of the individual values. For a only three measurements SDs are invalid, and almost never really reflect the distributions of the values.
We are also critical about this point, but other reviewers requested exactly this kind of statistics in the past. We would prefer to keep the figures in this format.
5. Please provide a very approximate estimate of how much H2A.Z and pol II are left after RNAi - the existing Westerns are enough to do this.
We cannot monitor Pol II expression levels because we don't have an antibody but considering the strong phenotype, we assume that there is a substantial reduction of protein expression. We assume the same for H2A.Z expression after RNAi.
6. Figure 8: The authors state that not all cells had chromosome condensation. Some quantitation is needed for this. How many showed it in + and - RNAi? Suggest sources of the variation.
Quantification of chromatin condensation is very difficult. For example, the pattern looks already different dependent of the section of the nucleus. To get a better impression of the phenotype, we provided a panel of different pictures for all conditions as supplementary figure S1.
7. Tables 1 and 2: provide data about localization from Tryp-Tag.
We added this information to tables 1 & 2.
8. Line 118-119. Please delete the phrase "one of the earliest-branching eukaryotic lineages". ALL eukaryotes belong to "early-branching" lineages. See 10.1101/cshperspect.a016147 or even 10.1073pnas.0807880106. As an alternative, authors could replace this phrase with an accurate statement.
We deleted the phrase.
9. There is no real need to defend use of luciferase as a reporter (lines 262-264) although and citing just one paper doesn't demonstrate "extensive" use. It is really good for looking at initial gene expression. However it is really not appropriate to use it to measure decreases in mRNA since luciferase protein is, if wild-type, imported into the glycosomes and probably has quite a long half-life. Since this is - as the authors admit - a very indirect measurement, I think these measurements should be deleted. They do not really add to the paper since the mRNA measurements, which the authors also have done, are much better.
We changed the manuscript accordingly but would prefer to keep the data in the manuscript because they are perfectly complementary to the mRNA measurements.
10. Line 398 - the lack of an effect on SLRNA is surprising and deserves a bit more comment. Could it be that the very particular pol II intiation complex that transcribes this - with its associated transcription factors - enables it to continue without SWR1? (Refer to relevant literature.) Also this is a steady-state level, and loss of transcription might be compensated by the fact that there is less precursor available, so the SLRNA won't be consumed as fast as usual.
We agree that this is surprising. Unfortunately, we do not have an explanation besides of what we already mention in the discussion. We are not aware of additional literature that might help to explain this phenomenon.

Reviewer #2: Concerns
1) As stated in the title, the authors claim to have characterized 'A novel SNF2 ATPase complex'. However, co-IP experiments alone are insufficient because [reciprocal] co-IP/-purification alone is not a stringent enough criterion for this claim (see also point 2). There is no additional evidence that these proteins do form a complex, meaning a stable entity, in trypanosomes. For example, the authors present a putative complex in Figure 1A (modeled according to the yeast counterpart) which probably has a combined molecular mass of several hundred kDa or more. If this complex does indeed exist, the authors need to present evidence (sizing column, native gel, sedimentation, etc) that some of the co-IPed proteins migrate together at the expected complex size. The same holds true for the HAT2 complex.

*We tried to provide these data, unfortunately, without success. We do not have antibodies for any components of the SWR1 complex, hence, we must work with HA-tagged proteins. However, these proteins are not detectable by Western blot analysis in whole cell lysates due to low expression levels. We only get weak signals after IP with lysates from at least10*8 cells. Hence, we don't think we can get enough material for gel filtration assays from a reasonable cell culture volume. We also tried to elute IPed material with HA peptides for native gel electrophoresis followed by silver staining but couldn't detect any proteins. Hence, we believe that it would be necessary to scale up the cell culture volumes substantially, to generate new cell lines and to establish the required methods in collaboration with our biochemistry department.*

However, we are not sure if this is necessary for this study. The composition of the complex has also been described by another group (Staneva et al-, (2021) bioRxiv) but without functional analysis. We believe it is necessary to study the function of conserved protein (complexes) in trypanosomes, because they often acquire novel (additional) functions. For example, we described that the conserved histone methyltransferase DOT1 is involved in novel functions (replication regulation) and exhibits trypanosome-specific protein interactions (with an RNaseH2 complex). We also describe novel components of the trypanosome SWR1 complex (eg. the putative SET methyltransferase). Unfortunately, all attempts of genetic manipulation (three independent RNAi constructs, N- and C-terminal in situ tagging, gene deletion) to characterize this component failed (we tested more than 200 clonal cell lines).

The function of the trypanosome SWR1 complex appears to be conserved. But this should also be communicated to the scientific community.

2) The presented mass spec data of the co-IPs are problematic. It is highly unusual to detect enrichment of only ~15 proteins in a co-IP with moderate E values. There are numerous reports in the field in which proteins were isolated in multiple steps in much higher purity than can be achieved by a single IP. Nevertheless, in these reports often more than a hundred proteins co-purified with enrichment and e-values that meet the criterions used here, and most of them did not define a complex. Hence, the data set presented appears to be highly selective. While I am not sure about PLoS Pathogens policy, mass spec data including "raw files" of all identified proteins, i.e. prior to the authors' selection, should be deposited in public repositories such as PRIDE. Apparently, this has not been done and, therefore, it is impossible to assess whether the presented proteins in the tables were selected in an unbiased manner. In addition, the authors claim to have carried out "quantitative proteomics" (line 130). I assume they used label-free quantification; if so, this should be better specified in the methods.

We apologize that we haven't done that before submission of the manuscript. As mentioned above, access to the MS proteomics data and the ChIPseq metadata have been added to the material and methods section. We also added information to the material & methods section.

3) The authors' claim of a novel SWR1 (and HAT2 complex) is based on co-IP of proteins that are either conserved only in trypanosomes or not part of the corresponding complex in yeast. However, in addition to not using a second criterion for complex formation, the authors fail to show that these genes/proteins have H2A.Z-specific functions. How do they justify 'novel'? Size exclusion chromatography and discuss

As described above (1), we tried to provide these data. The characterization of novel components (eg. the putative SET methyltransferase and additional BDFs) will be in the focus of future projects. However, this requires substantial more time than allowed for this revision and additional funding.

4) Figures 5-7: luciferase (protein-based) reporter assays and steady-state mRNA quantification are indirect methods to assay for a transcription defect. Transcriptional activity is best measured by labeling and quantifying newly synthesized pre-m/rRNA.

This is true, but considering that the complex is apparently functionally conserved in T. brucei and that all our data (and published data) are in line with a function in regulating transcriptional activity, we don't think that this will add very much to the manuscript.

5) It should be mentioned in the results section how the proteins were tagged and which antibody and protein solution was used for the IP since this is critical information to evaluate co-IP & mass spec results. In addition, it would be reassuring if the authors showed immunoblots, demonstrating IP efficiency. (Probably, protein staining would not reveal enrichment).
This information is already provided in the material & methods section (HA 12CA5 mouse monoclonal IgG (Sigma Aldrich) or anti-Ty1 (BB2) mouse monoclonal IgG) including reference. The cell lines are described in the supplemental methods. IP efficiency cannot be evaluated because the tagged proteins are not detectable in the input material.
6) Figure 8: is there a way to quantify condensed chromatin in un- versus treated cells to make this more convincing?
As mentioned above, this is very difficult and we would prefer to provide a larger set of EM pictures to the reader to get a better impression of the phenotype.

Reviewer #3: None noted

Part III – Minor Issues: Editorial and Data Presentation Modifications
Please use this section for editorial suggestions as well as relatively minor modifications of existing data that would enhance clarity.

Reviewer #1:
Line 430 - it would be interesting to look at other eukaryotic supergroups too. What is known about plants?
There is not as much literature about this complex in plants compared to yeast and mammals but is quite conserved for example in Arabidopsis.
line 46: elementary knowledge, delete. rest of paragraph can be shortened.
We deleted line 46.
line 64: Should be “proteins of all subfamilies”.
We changed the manuscript accordingly.
Line 103: why “eponymous”? Also, need a comma after ATPase and another “which” instead of “that”. (“Which” describes, “that” defines; they can’t be interchanged.)
We changed the manuscript accordingly.
Lines 105- onwards - refer to Fig 1. I found Fig1 difficult to read - please make all the text at least the same size as the largest in the external labels, it appears to me that the labels “within” the proteins are currently smaller.
We changed the figure accordingly.
Last paragrpah of the introduction is a repetition of the abstract, and could be shortened just to state the aims.
Table 1 - what does the asterisk mean? Please explain briefly in the Legend how the NES scores can be interpreted (are they explained more in the MEthods?) Also mention how were the p-values obtained, and what the nerichemtn was relative to (i.e. what the control was). Decide whether or not to capitalise the first letter of the Annotation.
We changed the table accordingly (asterisk deleted). NES interpretation was added to the legend in table2
Fig 5 - unless you measured the copy-number, delete “single” from the legend since we can never be sure there was only one integration. (Same for FIg 6.) Labels on the graphs in Fig 5 are microscopic - and the superscripts can be replaced if you use “log cell number” as the label for the y-axis.
We changed the figures accordingly.
Fig 7: replace hairlines in “B” with slightly thicker lines.
We changed the figure accordingly.
Line 539: missing paragraph mark.

Lines 364-372 - I think these belong in the REsults section.
We deleted this part of the discussion.
Fig S7 - should be “anucleate cells”. or better just “cells without nuclei”.
We changed the figure accordingly.
Line 387 - I believe that PLoS doesn’t allow the phrase “data not shown”. You can get around this by instead writing “preliminary results suggested that....”.
We deleted this part of the discussion.
Line 399: “spliced leader mRNA that appears to be mainly unaffected by TbSWR1 depletion” implies that there is other spliced leader RNA that IS affected. REplace “that” with “which”.
We changed the text accordingly.

Reviewer #2: point 5 above is a minor issue.

Reviewer #3: Line 188-189: Do the five proteins identified in the RuvB2 co-IP that are not part of the TbSWR1 complex have any similarity to the components of the yeast INO80 (or any other chromatin modification) complex?
They don’t have any similarity to INO80 proteins. RuvB2 together with Cbf5p and SHQ1 are associated with ‘small nucleolar ribonucleoprotein (snoRNP) biogenesis.
Line 246: Please provide a reference for the Co-IP experiments with HAT2.
We changed the text accordingly.
Line 224-335: What methods were used to exclude the presence of a DAMP-1 homologue in T. brucei?
We did not completely exclude the presence of a DAMP-1 domain, Phyre2 homology modelling indicated the presence of a DAMP-1 domain in TbSWRC4, but this could not be confirmed by BLAST analysis.
Line 427: Is it possible that H2A.Z does not need to be removed from chromatin, since transcription initiation is essentially constitutive in trypanosomatids?
This is a very good point. We changed the discussion accordingly.
Line 428-429: Jensen et al (mSphere, 2021) recently described a putative chromatin remodeling complex associated with JBP3 in Leishmania, orthologues of which are present in T. brucei.
Would it be possible to combine Tables 1 and 2?
This is difficult. The columns are different because the content is not the same (summary of the RuvB co-IP versus characteristics of the components). The list of components is also different.
Lines 1026 and 1031: “bolt” should be replaced with “bold” in the Figure 1legend.
We changed the text accordingly.
Figure 3: As currently shown, the depletion of H2A.Z at TSSs is not very convincing. I suggest that using more contrasting colors and/or normalizing to WT might be helpful, as would adding cartoons representing the different PTUs in panel A.
We changed the colors in the figure accordingly. We usually use uninduced cells as a control and don’t have ChIP data of WT cells. We prefer not to include a cartoon with PTUs, which is not very clear at this size.
