# Supplementary material for: A novel SNF2 ATPase complex in Trypanosoma brucei with a role in H2A.Z-mediated chromatin remodelling
Source: PLoS Pathog. 2022 Jun 8;18(6):e1010514. doi: 10.1371/journal.ppat.1010514 (PMC9236257; doi:10.1371/journal.ppat.1010514)
Supplement: Attachment — Submitted filename: response to reviewer2.pdf [file ppat.1010514.s005.pdf]

Wuerzburg, March 7th 2022

Dear Editor

Thank you for your letter and the referee's comments on our manuscript. We were happy to learn that the referees' comments on our revised manuscript were so positive. All major issues were addressed. There was only one minor request from reviewer #1 (Add individual data points in all graphs). We changed all figures accordingly. We also addressed all points raised by the editors and hope that our manuscript is now suitable for publication in *PLoS Pathogen*.

Sincerely,

Falk Butter & Christian Janzen

Dear Prof Butter and Prof Janzen,

Thanks for your revised manuscript. Before we can make a final decision, please can you address the following outstanding issues:

1. Adjust the data presentation as per reviewer-1's comment (part-III)

We changed all figures accordingly.

2. Clarify your response (2.1) to reviewer-2's comments on confirmatory data for the putative novel complex. You mentioned that you've run native gels following coIP, but not been able to detect protein by silver staining. Have you been able to detect the complex on these native gels using anti-tag antibodies? Are you able to localise (or co-localise) the tagged proteins, rather than citing TrypTag procyclic data?

Unfortunately, we were never able to detect any components of the complex by Western blotting of native gels. Co-localization studies were not possible because we don't have antibodies specific to all components of the complex. Tagging of proteins could be an alternative but this would simply be a repetition of experiments already done by the TrypTag consortium. We don't think this would generate any new insights for our manuscript.

In addition to the above (and the specific reviewer comments below), please can you address the following:

3. Provide evidence (northern or qRT-PCR) for SWR1 and Bdf3 knockdown

We believe that Norther or qRT-PCR are not suitable methods to provide quantitative data on SWR1 and Bdf3 knockdown. The problem is that an internal standard RNA is needed for normalization of the data. Since we are generating a global transcription defect by SWR1 RNAi, there is no suitable RNA for normalization because all RNAs are affected by SWR1 and Bdf3 knockdown. To circumvent this problem, we tried to quantify the decrease of SWR1 and Bdf3 mRNA by RNA FISH. This worked for SWR1 (data provided as supplementary figure) but not for Bdf3 most likely due to low abundance of the mRNA.

4. Update the Staneva ref to reflect its recent publication in Genome Research

We changed the manuscript accordingly.

5. Remove SK from the acknowledgements (already an author)

We changed the manuscript accordingly.

6. Two versions of the figures were included in your revised submission - please ensure that only the definitive versions are included in future; one version of figures 5 and 7 includes the correct sub-panel labeling, the other doesn't

We provided new figures.

7. In the chart legends, please be consistent in the use of 'light blue' or 'turquoise'; if using the latter, please check the spelling (S9)

We changed the manuscript accordingly.

Reviewer Comments (if any, and for reference):

Reviewer's Responses to Questions

Part I - Summary

Please use this section to discuss strengths/weaknesses of study, novelty/significance, general execution and scholarship.

Reviewer #1: In this paper the authors have identified trypanosome chromatin remodelling complexes in the T. brucei by mass spectrometry, and shown that depletion of a component leads to a decrease in steady-state mRNA and deposition of histone H2AZ.

Reviewer #2: The revised manuscript by Vellmer et al. has met several formal requirements of reviewers such as data submission to public data banks and changes in text. However, key experimental critiques could not be addressed. In general, the authors were unable to detect subunits of their complexes by tagging and to ablate the genes of putative trypanosome-specific subunits,

rendering their [functional] participation in SNF2 and HAT2 complexes weakly supported. As detailed in the previous review, this study is mainly confirmatory in nature.

In sum, this study clearly shows that, as in other organisms, SWR1 is essential for H2A.Z deposition and it suggests that SWR1 ablation leads to a transcriptional defect. Although the latter was shown in a rather indirect manner (reporter protein), it is the most important aspect of the study.

However, the conceptual/experimental weaknesses of the study predominate and are reiterated here in short:

(i) Annotation of the SWR1 homolog in trypanosomes would have been straightforward by a simple BLAST search; it did not need the elaborate bioinformatics analysis laid out here.
We simply tried to identify a SWR1 homolog by searching for characteristic motifs. This is not really a "elaborate bioinformatic analysis".

(ii) The putative trypanosome SNF2 complex depicted in Fig 1 remains extremely speculative without a second criterion and any functional data supporting it. Given that dozens of gene expression factors have been readily detectable in various laboratories, it is difficult to understand why the authors were not able to detect them.
We don't understand what the reviewers means with "dozens of gene expression factors" that have been detected in other laboratories. Maybe the reviewer is not aware of the fact that T. brucei doesn't posses specific transcription factors.

(iii) "Novel" features of the SNF2 complex (in the title!) are solely based on bioinformatics of co-precipitated proteins. There is no functional data supporting any of the authors' claims. Again, the assertion of the authors that they tried in vain to ablate genes of trypanosome-specific subunits is not encouraging and speaks against the relevance of the encoded proteins in the two complexes.
As mentioned already in the previous letter, we tried intensively to learn more about the function of novel factors of the complex by genetic manipulation. We don't understand why the failure of genetic manipulation speaks necessarily against a function within the complex.

(iv) Knockdown data are provided only for SWR1 which is a highly conserved protein with known function as well as BDF3 who has already been described and linked to H2.AZ. These functional data are mainly confirmatory in nature (Why could the authors knock down these factors successfully? Is it possible that the other knockdowns did not reveal anticipated results?).
The only other component we tried to knock down was the putative histone methyltransferase. As mentioned above, this didn't work.

Finally, the reference to another bioRxiv paper lacking functional data is not helpful.
This work (now published in Genome Research) confirms our data.

Reviewer #3: The authors have responded to most of the comments from the previous review and the manuscript is now ready for publication.

Part II – Major Issues: Key Experiments Required for Acceptance
Please use this section to detail the key new experiments or modifications of existing experiments that should be absolutely required to validate study conclusions.

Generally, there should be no more than 3 such required experiments or major modifications for a "Major Revision" recommendation. If more than 3 experiments are necessary to validate the study conclusions, then you are encouraged to recommend "Reject".

Reviewer #1: None
Reviewer #2: This was detailed in the original critique. The authors claim that they have tried to do the key experiments without success.
Reviewer #3: None

Part III – Minor Issues: Editorial and Data Presentation Modifications
Please use this section for editorial suggestions as well as relatively minor modifications of existing data that would enhance clarity.
Reviewer #1: In their responses the authors expend a lot of effort in saying why they don't want to change most aspects of the manuscript. I have just one request:

"We are also critical about this point, but other reviewers requested exactly this kind of statistics in the past. We would prefer to keep the figures in this format." If you are critical then change it! Just because reviewers make mistakes there is no need to follow their advice. I have had no trouble whatsoever publishing graphs with individual values, there is an excellent argument for doing so and it is becoming increasingly common. If you insist on keeping bar graphs with invalid standard deviations, at least superimpose the individual values as clearly visible spots.
We changed the figures accordingly.

Reviewer #2: at this point, not relevant.
Reviewer #3: None
